# The genome sequence of Risso’s dolphin, *Grampus griseus* (G.Cuvier, 1812) (Artiodactyla: Delphinidae)

**DOI:** 10.12688/wellcomeopenres.24752.1

**Published:** 2025-08-11

**Authors:** Nicholas J. Davison, Phillip A. Morin, Wellcome Sanger Institute Tree of Life Management, Samples and Laboratory team, Wellcome Sanger Institute Scientific Operations – Sequencing Operations, Wellcome Sanger Institute Tree of Life Core Informatics team, Tree of Life Core Informatics collective, Darwin Tree of Life Consortium

**Affiliations:** 1 Scottish Marine Animal Stranding Scheme, University of Glasgow, Glasgow, Scotland, UK; 2 Southwest Fisheries Science Center, NOAA, La Jolla, California, USA

**Keywords:** Grampus griseus, Risso's dolphin, genome sequence, chromosomal, Artiodactyla

## Abstract

We present a genome assembly from a male specimen of *Grampus griseus* (Risso’s dolphin; Chordata; Mammalia; Artiodactyla; Delphinidae). The assembly contains two haplotypes with total lengths of 2 667.07 megabases and 2 403.96 megabases. Most of haplotype 1 (89.46%) is scaffolded into 23 chromosomal pseudomolecules, including the X and Y sex chromosomes. Haplotype 2 was assembled to scaffold level. The mitochondrial genome has also been assembled, with a length of 16.39 kilobases.

## Species taxonomy

Eukaryota; Opisthokonta; Metazoa; Eumetazoa; Bilateria; Deuterostomia; Chordata; Craniata; Vertebrata; Gnathostomata; Teleostomi; Euteleostomi; Sarcopterygii; Dipnotetrapodomorpha; Tetrapoda; Amniota; Mammalia; Theria; Eutheria; Boreoeutheria; Laurasiatheria; Artiodactyla; Whippomorpha; Cetacea; Odontoceti; Delphinidae; *Grampus*; *Grampus griseus* (G.Cuvier, 1812) (NCBI:txid83653)

## Background

Risso’s dolphin ( *Grampus griseus*) is a large dolphin (adult size 3.6–4 m) commonly found in warm temperate and tropical waters globally, concentrating in deep offshore waters along continental slopes. They are included in the group of dolphin species commonly referred to as “blackfish”, which include pilot whales ( *Globicephala* spp.), false killer whales ( *Pseudorca crassidens*), melon-headed whales ( *Peponocephala electra*), killer whales ( *Orcinus* spp.) and pygmy killer whales ( *Feresa attenuata*) ( Hartman, 2018).

Although Risso’s dolphins most often travel in groups of 10 to 30 individuals, aggregations of several thousand have been observed off California. They concentrate along the upper continental slope and around steep shelf-edge areas ( Hartman, 2018), feeding primarily on deep-water squid and octopus. In some regions there is evidence of sex-stratified social organisation and strong site fidelity.

While listed as Least Concern globally by the IUCN, the subpopulation of Risso’s dolphins in the Mediterranean is considered Endangered, with a decreasing population due mainly to fisheries bycatch (IUCNredlist.org, consulted 20 February 2025). Other threats include oceanic noise, chemical and plastic pollution, and climate change.

**Figure 1.**
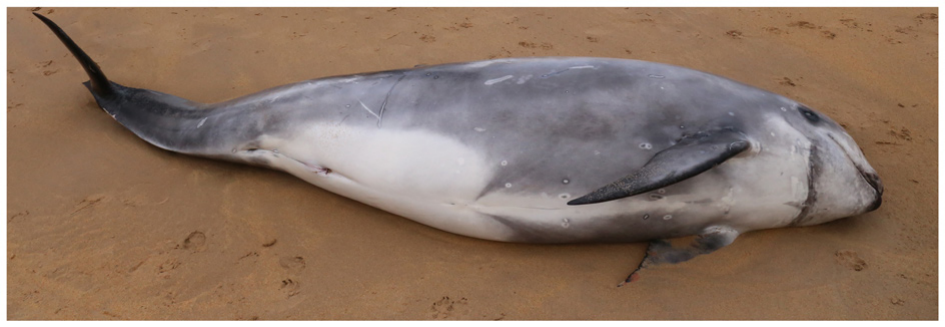
Photograph of the *Grampus griseus* (mGraGri1) carcass from which samples were taken for genome sequencing (photo credit Nick Davison).

As part of the Darwin Tree of Life Project – which aims to generate high-quality reference genomes for all named eukaryotic species in Britain and Ireland to support research, conservation, and the sustainable use of biodiversity – we present a chromosomally complete genome sequence for the Risso’s dolphin, *Grampus griseus*. This genome was assembled using the Tree of Life pipeline from a specimen collected in Waters of Philorth, Fraserbrugh, Aberdeenshire, Scotland, United Kingdom ( Figure 1).

## Methods

### Sample acquisition

The specimen used for genome sequencing was an adult male *Grampus griseus* (specimen ID SAN00002610, ToLID mGraGri1; Figure 1), collected from Waters of Philorth, Fraserbrugh, Aberdeenshire, Scotland, United Kingdom (latitude 57.6758, longitude -1.9708) on 2022-02-15. The specimen was collected and identified by Nick Davison (Scottish Marine Animal Stranding Scheme, University of Glasgow). Sample metadata was collected in line with the Darwin Tree of Life project standards described by Lawniczak *et al*. ( Lawniczak *et al.*, 2022).

### Nucleic acid extraction

Protocols for high molecular weight (HMW) DNA extraction developed at the Wellcome Sanger Institute (WSI) Tree of Life Core Laboratory are available on protocols.io ( Howard *et al.*, 2025). The mGraGri1 sample was weighed and triaged to determine the appropriate extraction protocol. Tissue from the lung was homogenised by cryogenic disruption using the Covaris cryoPREP ^®^ Automated Dry Pulverizer.

HMW DNA was extracted using the Automated MagAttract v2 protocol. DNA was sheared into an average fragment size of 12–20 kb following the Megaruptor®3 for LI PacBio protocol. Sheared DNA was purified by manual SPRI (solid-phase reversible immobilisation). The concentration of the sheared and purified DNA was assessed using a Nanodrop spectrophotometer and Qubit Fluorometer using the Qubit dsDNA High Sensitivity Assay kit. Fragment size distribution was evaluated by running the sample on the FemtoPulse system.

RNA was extracted from lung tissue of mGraGri1 in the Tree of Life Laboratory at the WSI using the RNA Extraction: Automated MagMax™ *mir*Vana protocol. The RNA concentration was assessed using a Nanodrop spectrophotometer and a Qubit Fluorometer using the Qubit RNA Broad-Range Assay kit. Analysis of the integrity of the RNA was done using the Agilent RNA 6000 Pico Kit and Eukaryotic Total RNA assay.

### PacBio HiFi library preparation and sequencing

Library preparation and sequencing were performed at the WSI Scientific Operations core.

***Library prep for PacBio LI***

Samples with an average fragment size greater than 8 kb and total mass exceeding 400 ng were eligible for the low-input SMRTbell Prep Kit 3.0 protocol (Pacific Biosciences, California, USA), depending on genome size and required sequencing depth. Libraries were prepared using the SMRTbell Prep Kit 3.0 according to the manufacturer’s instructions. The kit includes reagents for end repair/A-tailing, adapter ligation, post-ligation SMRTbell bead clean-up, and nuclease treatment. Size selection and clean-up were performed using diluted AMPure PB beads (Pacific Biosciences). DNA concentration was quantified using a Qubit Fluorometer v4.0 (ThermoFisher Scientific) and the Qubit 1X dsDNA HS assay kit. Final library fragment size was assessed with the Agilent Femto Pulse Automated Pulsed Field CE Instrument (Agilent Technologies) using the gDNA 55 kb BAC analysis kit.

The sample was sequenced on a Revio instrument (Pacific Biosciences). The prepared library was normalised to 2 nM, and 15 μL was used for making complexes. Primers were annealed and polymerases bound to generate circularised complexes, following the manufacturer’s instructions. Complexes were purified using 1.2X SMRTbell beads, then diluted to the Revio loading concentration (200–300 pM) and spiked with a Revio sequencing internal control. The sample was sequenced on a Revio 25M SMRT cell. The SMRT Link software (Pacific Biosciences), a web-based workflow manager, was used to configure and monitor the run and to carry out primary and secondary data analysis.

### Hi-C preparation

***Sample preparation and crosslinking***

The Hi-C sample was prepared from 20–50 mg of frozen lung tissue from the mGraGri1 sample using the Arima-HiC v2 kit (Arima Genomics). Following the manufacturer’s instructions, tissue was fixed and DNA crosslinked using TC buffer, giving a final formaldehyde concentration of 2%. The tissue was homogenised using the Diagnocine Power Masher-II. Crosslinked DNA was digested with a restriction enzyme master mix, biotinylated, and ligated. Clean-up was performed with SPRISelect beads before library preparation. DNA concentration was measured with the Qubit Fluorometer (Thermo Fisher Scientific) and Qubit HS Assay Kit. The biotinylation percentage was estimated using the Arima-HiC v2 QC beads.

***Hi-C library preparation and sequencing***

Biotinylated DNA constructs were fragmented using a Covaris E220 sonicator and size selected to 400–600 bp using SPRISelect beads. DNA was enriched with Arima-HiC v2 kit Enrichment beads. End repair, A-tailing, and adapter ligation were carried out with the NEBNext Ultra II DNA Library Prep Kit (New England Biolabs), following a modified protocol where library preparation occurs while DNA remains bound to the Enrichment beads. Library amplification was performed using KAPA HiFi HotStart mix and a custom Unique Dual Index (UDI) barcode set (Integrated DNA Technologies). Depending on sample concentration and biotinylation percentage determined at the crosslinking stage, libraries were amplified with 10–16 PCR cycles. Post-PCR clean-up was performed with SPRISelect beads. Libraries were quantified using the AccuClear Ultra High Sensitivity dsDNA Standards Assay Kit (Biotium) and a FLUOstar Omega plate reader (BMG Labtech).

Prior to sequencing, libraries were normalised to 10 ng/μL. Normalised libraries were quantified again and equimolar and/or weighted 2.8 nM pools. Pool concentrations were checked using the Agilent 4200 TapeStation (Agilent) with High Sensitivity D500 reagents before sequencing. Sequencing was performed using paired-end 150 bp reads on the Illumina NovaSeq 6000.

### RNA library preparation and sequencing

Libraries were prepared using the NEBNext ^®^ Ultra™ II Directional RNA Library Prep Kit for Illumina (New England Biolabs), following the manufacturer’s instructions. Poly(A) mRNA in the total RNA solution was isolated using oligo(dT) beads, converted to cDNA, and uniquely indexed; 14 PCR cycles were performed. Libraries were size-selected to produce fragments between 100–300 bp. Libraries were quantified, normalised, pooled to a final concentration of 2.8 nM, and diluted to 150 pM for loading. Sequencing was carried out on the Illumina NovaSeq X to generate 150-bp paired-end reads.

### Genome assembly

Prior to assembly of the PacBio HiFi reads, a database of *k*-mer counts ( *k* = 31) was generated from the filtered reads using FastK. GenomeScope2 ( Ranallo-Benavidez *et al.*, 2020) was used to analyse the *k*-mer frequency distributions, providing estimates of genome size, heterozygosity, and repeat content.

The HiFi reads were assembled using Hifiasm in Hi-C phasing mode ( Cheng *et al.*, 2021; Cheng *et al.*, 2022), producing two haplotypes. Hi-C reads ( Rao *et al.*, 2014) were mapped to the primary contigs using bwa-mem2 ( Vasimuddin *et al.*, 2019) Contigs were further scaffolded with Hi-C data in YaHS ( Zhou *et al.*, 2023), using the --break option for handling potential misassemblies. The scaffolded assemblies were evaluated using Gfastats ( Formenti *et al.*, 2022), BUSCO ( Manni *et al.*, 2021) and MERQURY.FK ( Rhie *et al.*, 2020).

The mitochondrial genome was assembled using MitoHiFi ( Uliano-Silva *et al.*, 2023), which runs MitoFinder ( Allio *et al.*, 2020) and uses these annotations to select the final mitochondrial contig and to ensure the general quality of the sequence.

### Assembly curation

The assembly was decontaminated using the Assembly Screen for Cobionts and Contaminants ( ASCC) pipeline. TreeVal was used to generate the flat files and maps for use in curation. Manual curation was conducted primarily in PretextView and HiGlass ( Kerpedjiev *et al.*, 2018). Scaffolds were visually inspected and corrected as described by Howe *et al*. (2021). The manual corrections included 13 breaks and 67 joins. The curation process is documented at https://gitlab.com/wtsi-grit/rapid-curation. PretextSnapshot was used to generate a Hi-C contact map of the final assembly.

### Assembly quality assessment

The Merqury.FK tool ( Rhie *et al.*, 2020), run in a Singularity container ( Kurtzer *et al.*, 2017), was used to evaluate *k*-mer completeness and assembly quality for both haplotypes using the *k*-mer databases ( *k* = 31) computed prior to genome assembly. The analysis outputs included assembly QV scores and completeness statistics.

The genome was analysed using the BlobToolKit pipeline, a Nextflow implementation of the earlier Snakemake BlobToolKit pipeline ( Challis *et al.*, 2020). The pipeline aligns PacBio reads using minimap2 ( Li, 2018) and SAMtools ( Danecek *et al.*, 2021) to generate coverage tracks. Simultaneously, it queries the NCBI taxonomy to identify relevant BUSCO lineages and runs BUSCO ( Manni *et al.*, 2021). For the three domain-level BUSCO lineages, BUSCO genes are aligned to the UniProt Reference Proteomes database ( Bateman *et al.*, 2023) using DIAMOND blastp ( Buchfink *et al.*, 2021). The genome is divided into chunks based on the density of BUSCO genes from the closest taxonomic lineage, and each chunk is aligned to the UniProt Reference Proteomes database with DIAMOND blastx. Sequences without hits are chunked using seqtk and aligned to the NT database with blastn ( Altschul *et al.*, 1990). The BlobToolKit suite consolidates all outputs into a blobdir for visualisation. The BlobToolKit pipeline was developed using nf-core tooling ( Ewels *et al.*, 2020) and MultiQC ( Ewels *et al.*, 2016), with containerisation through Docker ( Merkel, 2014) and Singularity ( Kurtzer *et al.*, 2017).

## Genome sequence report

### Sequence data

**Figure 2.**
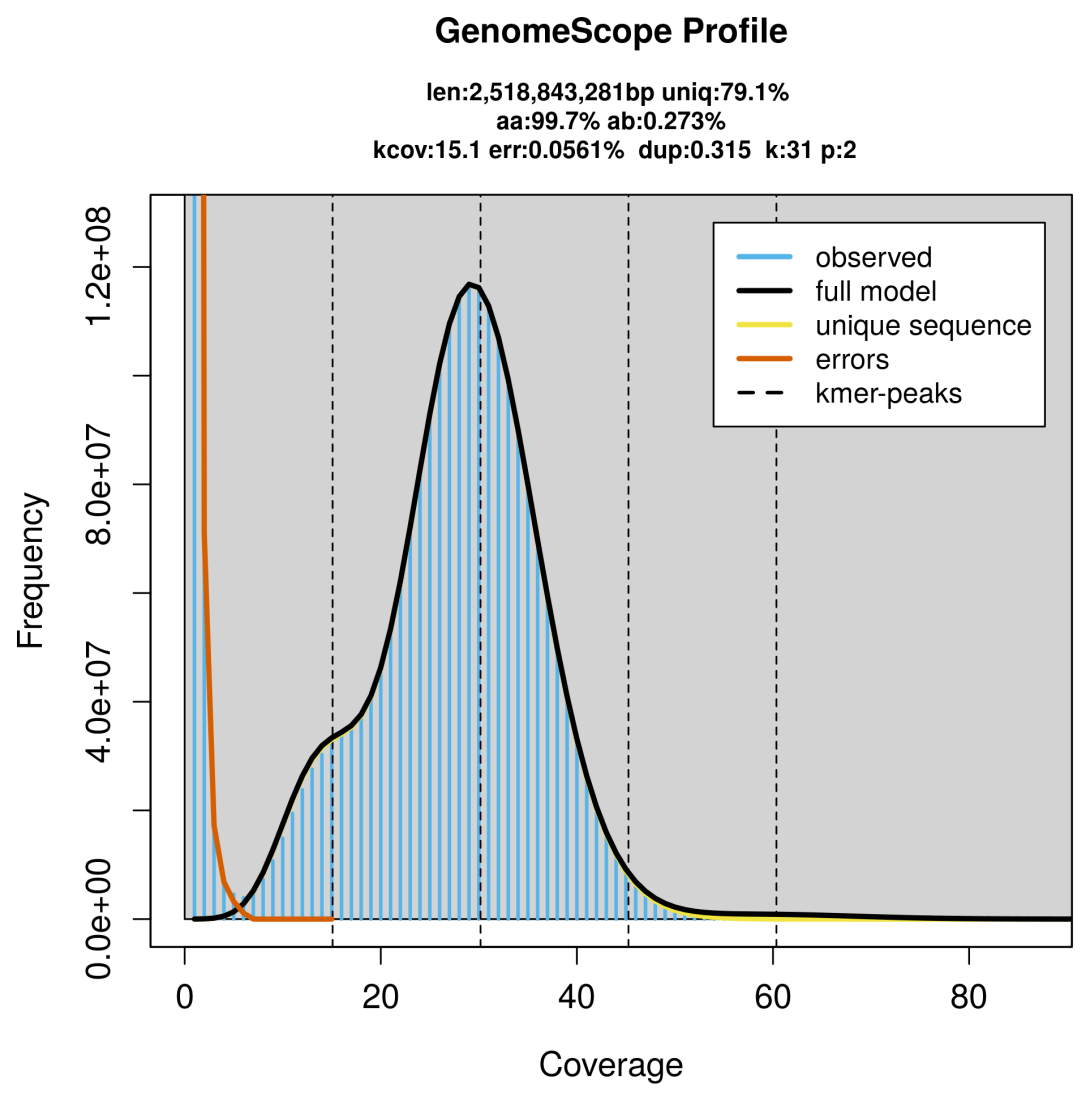
Frequency distribution of *k*-mers generated using GenomeScope2. The plot shows observed and modelled *k*-mer spectra, providing estimates of genome size, heterozygosity, and repeat content based on unassembled sequencing reads.

The genome of a specimen of *Grampus griseus* was sequenced using Pacific Biosciences single-molecule HiFi long reads, generating 77.58 Gb (gigabases) from 8.23 million reads, which were used to assemble the genome. GenomeScope analysis estimated the haploid genome size at 2 518.84 Mb, with a heterozygosity of 0.27% and repeat content of 20.98% ( Figure 2). These estimates guided expectations for the assembly. Based on the estimated genome size, the sequencing data provided approximately 30x coverage. Hi-C sequencing produced 440.58 Gb from 2 917.72 million reads, used to scaffold the assembly. RNA sequencing data were also generated and are available in public sequence repositories. Table 1 summarises the specimen and sequencing details.

**Table 1.** Specimen and sequencing data for BioProject PRJEB75720.

Platform	PacBio HiFi	Hi-C	RNA-seq
**ToLID**	mGraGri1	mGraGri1	mGraGri1
**Specimen ID**	SAN00002610	SAN00002610	SAN00002610
**BioSample (source individual)**	SAMEA111380541	SAMEA111380541	SAMEA111380541
**BioSample (tissue)**	SAMEA111380549	SAMEA111380549	SAMEA111380549
**Tissue**	lung	lung	lung
**Sequencing platform and model**	Revio	Illumina NovaSeq 6000	Illumina NovaSeq X
**Run accessions**	ERR13112083	ERR13132929	ERR13962515
**Read count total**	8.23 million	2 917.72 million	30.74 million
**Base count total**	77.58 Gb	440.58 Gb	4.64 Gb

### Assembly statistics

The genome was assembled into two haplotypes using Hi-C phasing. Haplotype 1 was curated to chromosome level, while haplotype 2 was assembled to scaffold level. The final assembly has a total length of 2 667.07 Mb in 1 145 scaffolds, with 1 138 gaps, and a scaffold N50 of 107.61 Mb ( Table 2).

**Table 2.** Genome assembly statistics.

Genome assembly	Haplotype 1	Haplotype 2
**Assembly name**	mGraGri1.hap1.1	mGraGri1.hap2.1
**Assembly accession**	GCA_964374135.1	GCA_964374185.1
**Assembly level**	chromosome	scaffold
**Span (Mb)**	2 667.07	2 403.96
**Number of chromosomes**	23	-
**Number of contigs**	2 283	2 135
**Contig N50**	3.11 Mb	3.24 Mb
**Number of scaffolds**	1 145	1 062
**Scaffold N50**	107.61 Mb	105.49 Mb
**Longest scaffold length (Mb)**	183.33	-
**Sex chromosomes**	X and Y	-
**Organelles**	Mitochondrial genome: 16.39 kb	-

**Figure 3.**
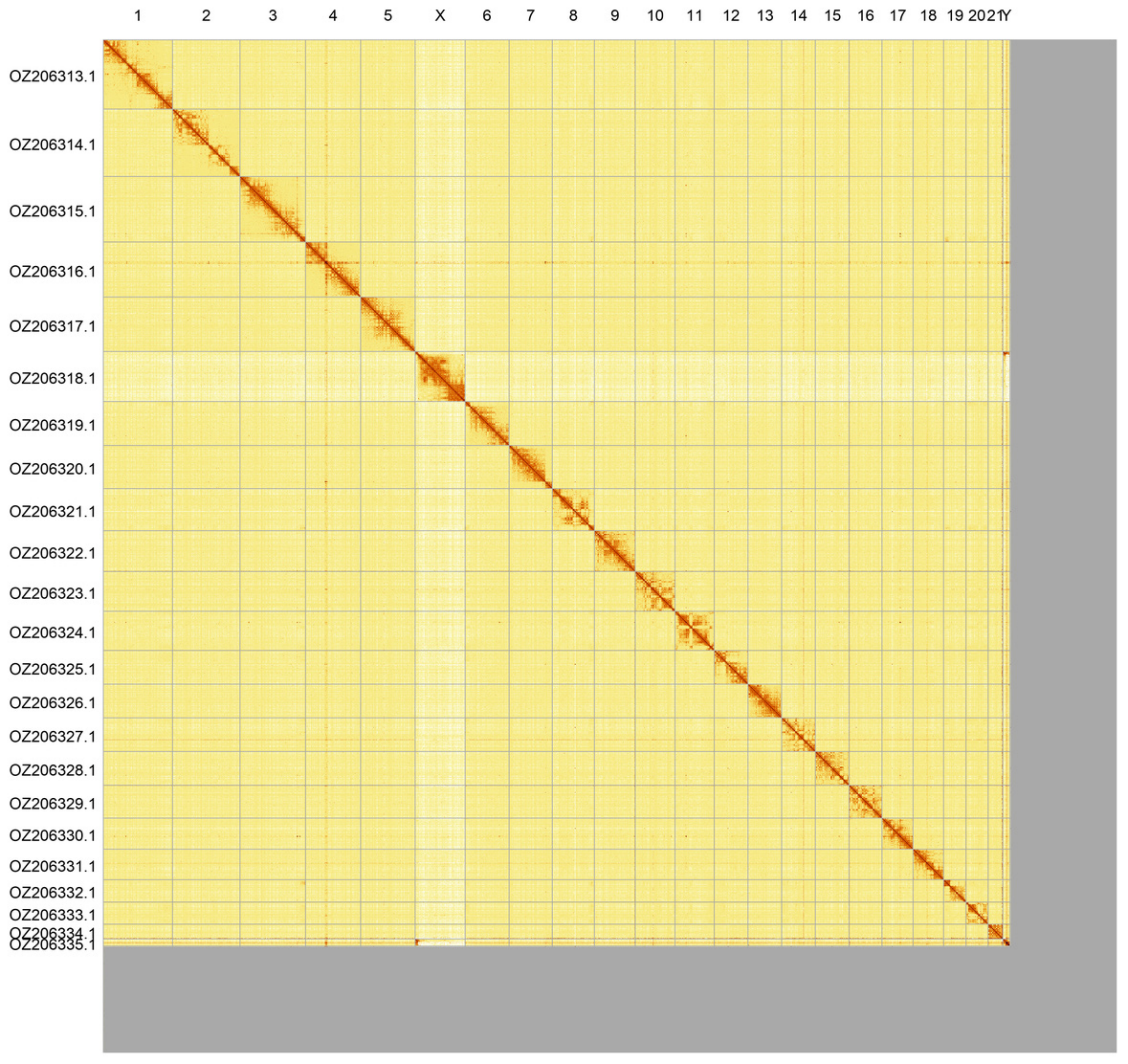
Hi-C contact map of the *Grampus griseus* genome assembly. Assembled chromosomes are shown in order of size and labelled along the axes. The plot was generated using PretextSnapshot.

Most of the assembly sequence (89.46%) was assigned to 23 chromosomal-level scaffolds, representing 21 autosomes and the X and Y sex chromosomes. These chromosome-level scaffolds, confirmed by Hi-C data, are named according to size ( Figure 3; Table 3).

**Table 3.** Chromosomal pseudomolecules in the haplotype 1 genome assembly of *Grampus griseus* mGraGri1.

INSDC accession	Molecule	Length (Mb)	GC%
OZ206313.1	1	183.33	42
OZ206314.1	2	177	41.50
OZ206315.1	3	173.06	41
OZ206316.1	4	144.35	39
OZ206317.1	5	143.81	39.50
OZ206319.1	6	115.64	42
OZ206320.1	7	114.16	40
OZ206321.1	8	110.54	40.50
OZ206322.1	9	107.61	40.50
OZ206323.1	10	104.34	43
OZ206324.1	11	103.03	41.50
OZ206325.1	12	88.97	43
OZ206326.1	13	88.78	39
OZ206327.1	14	88.75	46
OZ206328.1	15	88.51	41.50
OZ206329.1	16	86.70	43
OZ206330.1	17	81.58	40.50
OZ206331.1	18	80.08	39.50
OZ206332.1	19	58.78	45.50
OZ206333.1	20	58.44	46
OZ206334.1	21	39.16	42
OZ206318.1	X	131.18	40
OZ206335.1	Y	18.14	44
OZ206336.1	MT	0.02	39

The mitochondrial genome was also assembled. This sequence is included as a contig in the multifasta file of the genome submission and as a standalone record.

### Assembly quality metrics

**Figure 4.**
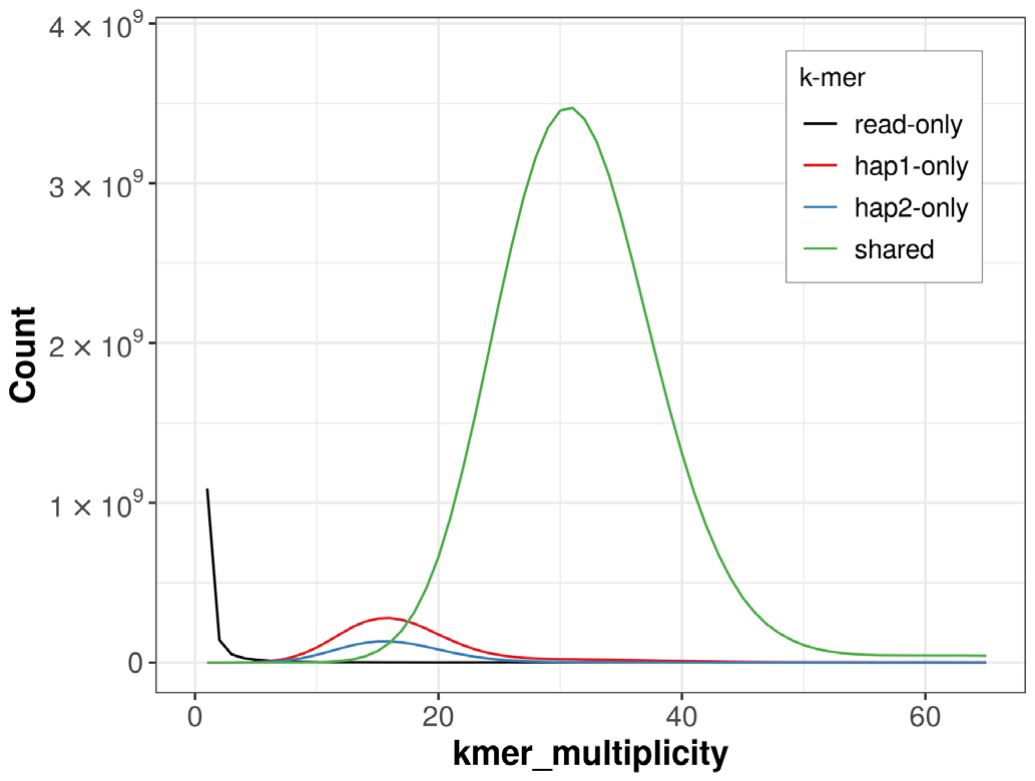
Evaluation of *k*-mer completeness using MerquryFK. This plot illustrates the recovery of *k*‐mers from the original read data in the final assemblies. The horizontal axis represents *k*‐mer multiplicity, and the vertical axis shows the number of *k*‐mers. The black curve represents *k*‐mers that appear in the reads but are not assembled. The green curve (the homozygous peak) corresponds to *k*‐mers shared by both haplotypes and the red and blue curves (the heterozygous peaks) show *k*‐mers found only in one of the haplotypes.

**Figure 5.**
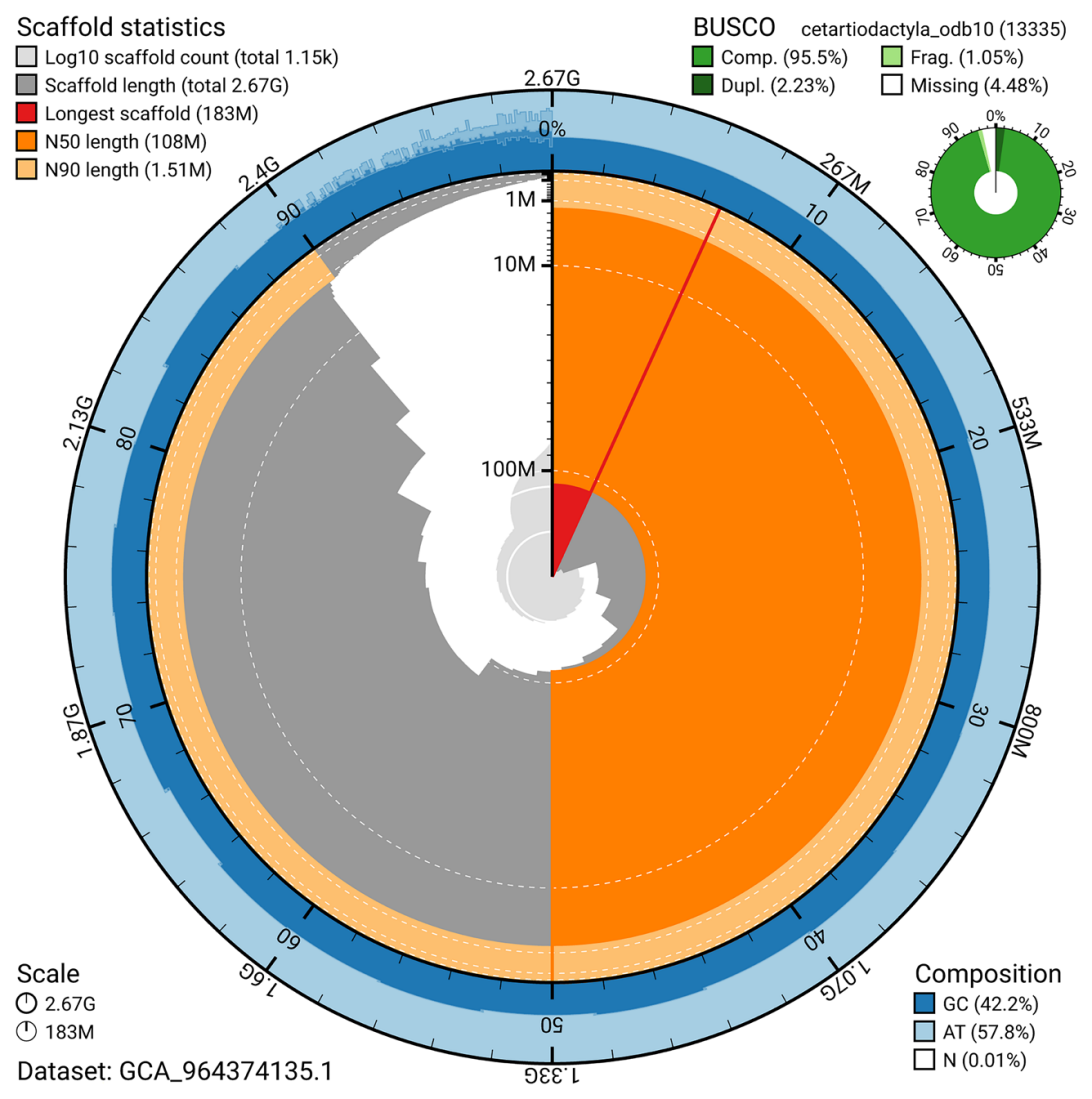
Assembly metrics for mGraGri1.hap1.1. The BlobToolKit snail plot provides an overview of assembly metrics and BUSCO gene completeness. The circumference represents the length of the whole genome sequence, and the main plot is divided into 1,000 bins around the circumference. The outermost blue tracks display the distribution of GC, AT, and N percentages across the bins. Scaffolds are arranged clockwise from longest to shortest and are depicted in dark grey. The longest scaffold is indicated by the red arc, and the deeper orange and pale orange arcs represent the N50 and N90 lengths. A light grey spiral at the centre shows the cumulative scaffold count on a logarithmic scale. A summary of complete, fragmented, duplicated, and missing BUSCO genes in the set is presented at the top right. An interactive version of this figure can be accessed on the BlobToolKit viewer.

**Figure 6.**
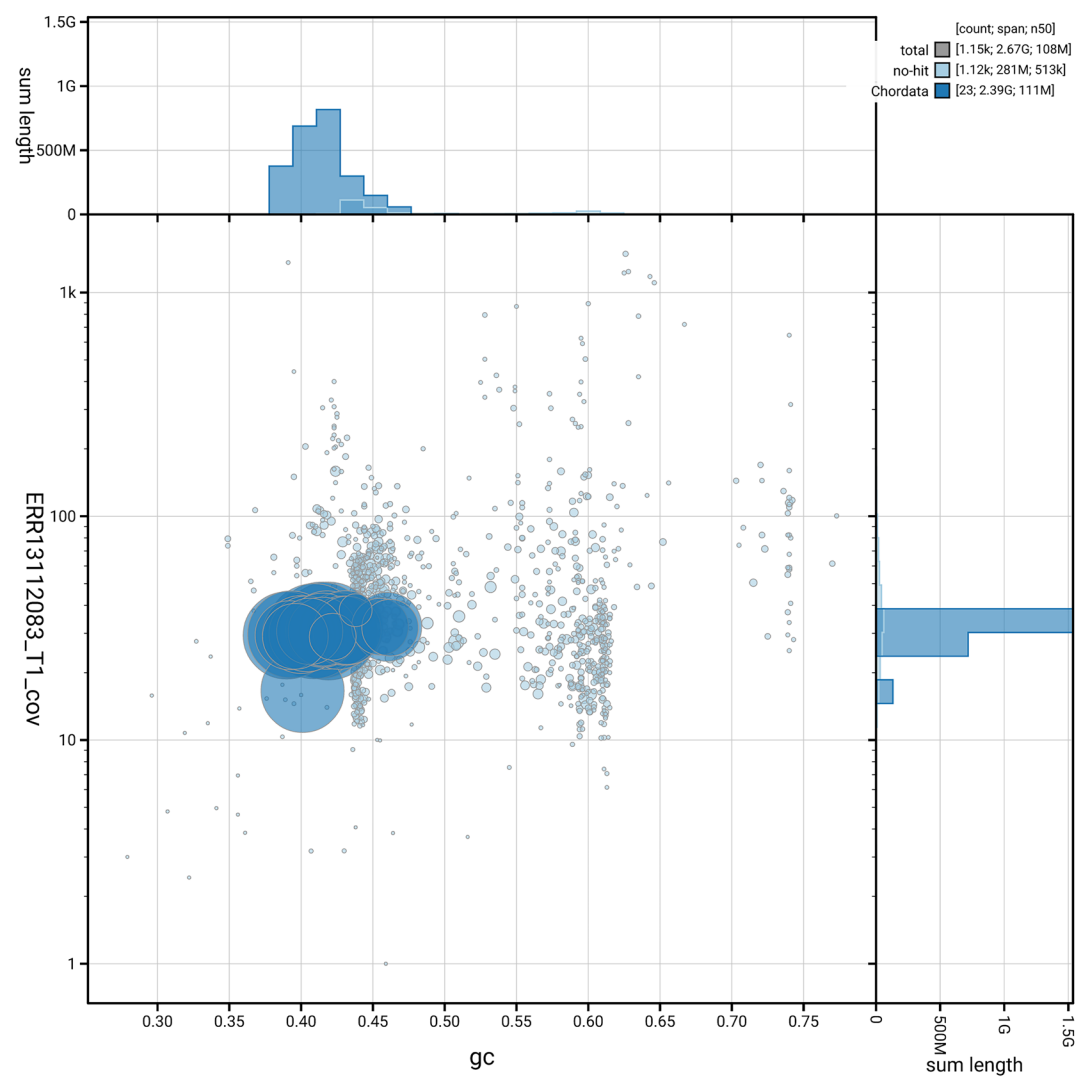
BlobToolKit GC-coverage plot for mGraGri1.hap1.1. Blob plot showing sequence coverage (vertical axis) and GC content (horizontal axis). The circles represent scaffolds, with the size proportional to scaffold length and the colour representing phylum membership. The histograms along the axes display the total length of sequences distributed across different levels of coverage and GC content. An interactive version of this figure is available on the BlobToolKit viewer.

For haplotype 1, the estimated QV is 65.5, and for haplotype 2, 65.6. When the two haplotypes are combined, the assembly achieves an estimated QV of 65.6. The *k*-mer completeness is 95.42% for haplotype 1, 90.44% for haplotype 2, and 99.76% for the combined haplotypes ( Figure 4). BUSCO v.5.5.0 analysis using the cetartiodactyla_odb10 reference set ( *n* = 13 335) identified 95.5% of the expected gene set (single = 93.3%, duplicated = 2.2%) for haplotype 1. The snail plot in Figure 5 summarises the scaffold length distribution and other assembly statistics for haplotype 1. The blob plot in Figure 6 shows the distribution of scaffolds by GC proportion and coverage for haplotype 1.

Table 4 lists the assembly metric benchmarks adapted from Rhie *et al.* (2021) the Earth BioGenome Project Report on Assembly Standards September 2024. The EBP metric, calculated for haplotype 1, is **6.8.Q65**.

**Table 4.** Earth Biogenome Project summary metrics for the *Grampus griseus* assembly.

Measure	Value	Benchmark
EBP summary (haplotype 1)	6.8.Q65	6.C.Q40
Contig N50 length	3.11 Mb	≥ 1 Mb
Scaffold N50 length	107.61 Mb	= chromosome N50
Consensus quality (QV)	Haplotype 1: 65.5; haplotype 2: 65.6; combined: 65.6	≥ 40
*k*-mer completeness	Haplotype 1: 95.42%; Haplotype 2: 90.44%; combined: 99.76%	≥ 95%
BUSCO	C:95.5%[S:93.3%‚D:2.2%]‚ F:1.0%‚M:3.4%‚n:13 335	S > 90%; D < 5%
Percentage of assembly assigned to chromosomes	89.46%	≥ 90%

### Wellcome Sanger Institute – Legal and Governance

The materials that have contributed to this genome note have been supplied by a Darwin Tree of Life Partner. The submission of materials by a Darwin Tree of Life Partner is subject to the **‘Darwin Tree of Life Project Sampling Code of Practice’**, which can be found in full on the Darwin Tree of Life website here. By agreeing with and signing up to the Sampling Code of Practice, the Darwin Tree of Life Partner agrees they will meet the legal and ethical requirements and standards set out within this document in respect of all samples acquired for, and supplied to, the Darwin Tree of Life Project.

Further, the Wellcome Sanger Institute employs a process whereby due diligence is carried out proportionate to the nature of the materials themselves, and the circumstances under which they have been/are to be collected and provided for use. The purpose of this is to address and mitigate any potential legal and/or ethical implications of receipt and use of the materials as part of the research project, and to ensure that in doing so we align with best practice wherever possible. The overarching areas of consideration are:

- Ethical review of provenance and sourcing of the material
- Legality of collection, transfer and use (national and international)

Each transfer of samples is further undertaken according to a Research Collaboration Agreement or Material Transfer Agreement entered into by the Darwin Tree of Life Partner, Genome Research Limited (operating as the Wellcome Sanger Institute), and in some circumstances other Darwin Tree of Life collaborators.

## Data Availability

European Nucleotide Archive: Grampus griseus (Risso’s dolphin). Accession number PRJEB75720. The genome sequence is released openly for reuse. The *Grampus griseus* genome sequencing initiative is part of the Darwin Tree of Life Project (PRJEB40665), Sanger Institute Tree of Life Programme (PRJEB43745), Cetacean Genomes Project (PRJNA1020146) and Vertebrate Genomes Project (PRJNA489243). All raw sequence data and the assembly have been deposited in INSDC databases. The genome will be annotated using available RNA-Seq data and presented through the Ensembl pipeline at the European Bioinformatics Institute. Raw data and assembly accession identifiers are reported in Table 1 and Table 2. Pipelines used for genome assembly at the WSI Tree of Life are available at https://pipelines.tol.sanger.ac.uk/pipelines. Table 5 lists software versions used in this study.

## Author information

Contributors are listed at the following links:

- Wellcome Sanger Institute Tree of Life Management, Samples and Laboratory team
- Wellcome Sanger Institute Scientific Operations – Sequencing Operations
- Wellcome Sanger Institute Tree of Life Core Informatics team
- Tree of Life Core Informatics collective
- Darwin Tree of Life Consortium
