# Supplementary material for: The genome sequence of Risso’s dolphin, Grampus griseus (G.Cuvier, 1812) (Artiodactyla: Delphinidae)
Source: Wellcome Open Res. 2025 Aug 11;10:431. [Version 1] doi: 10.12688/wellcomeopenres.24752.1 (PMC12476538; doi:10.12688/wellcomeopenres.24752.1)
Supplement: Peer review history

# Reviewer report: Reviewer response for version 1

**DOI:** 10.21956/wellcomeopenres.27269.r130297

**Published:** 2025-09-27

**Authors:** Luis A. Escobedo-Morales

**Affiliations:** 1 Universidad Nacional Autónoma de México, Mexico City, Mexico

The authors provide, for the first time, a high-quality, complete genome for a cetacean species of conservation interest due to its probable declining populations. Sequencing and assembly of the complete haplotype 1 at the chromosomal level, as well as most of haplotype 2, including the mitochondrial DNA genome, will serve as a reference for future population genomics and taxonomy.

 After a careful review of the article, I found it to be well-written, with clearly presented results and accessible data. I have no additional questions regarding the manuscript; it merits indexing and should move forward.

Are sufficient details of methods and materials provided to allow replication by others?

Yes

Is the rationale for creating the dataset(s) clearly described?

Yes

Are the datasets clearly presented in a useable and accessible format?

Yes

Are the protocols appropriate and is the work technically sound?

Yes

Reviewer Expertise:

Evolutionary biology, systematics, museomics

I confirm that I have read this submission and believe that I have an appropriate level of expertise to confirm that it is of an acceptable scientific standard.

# Reviewer report: Reviewer response for version 1

**DOI:** 10.21956/wellcomeopenres.27269.r132993

**Published:** 2025-09-24

**Authors:** Elisa Ramos

**Affiliations:** 1 Natural History Museum Basel, Basel, Switzerland; 2 Departement Umweltwissenschaften, Universitat Basel (Ringgold ID: 27209), Basel, Basel-Stadt, Switzerland

Davison and Morin et al present a high-quality, chromosomally complete genome assembly of *Grampus griseus* (Risso’s dolphin), providing an important genomic resource for studies of an ecologically significant cetacean and the sole representative of its genus. The authors produced comprehensive and well-curated assemblies for haplotype 1 and the mitochondrial genome. This work represents a valuable contribution to comparative genomics, evolutionary biology, and conservation, and a great advance for cetacean research, given the species uniqueness. The methods section is generally clear and detailed, though it would be helpful if the authors could also add the following information:

1. Since the entire carcass was accessible and the genome was assembled from lung tissue, is additional material stored in a biobank or museum collection for future use?
2. Only haplotype 1 was assembled to the chromosome level. Given that haplotype 2 also appears not to be highly fragmented do the authors plan to enhance this assembly as well in the future?

Are sufficient details of methods and materials provided to allow replication by others?

Yes

Is the rationale for creating the dataset(s) clearly described?

Yes

Are the datasets clearly presented in a useable and accessible format?

Partly

Are the protocols appropriate and is the work technically sound?

Yes

Reviewer Expertise:

Vertebrate comparative genomics, speciation and adaptation genomics

I confirm that I have read this submission and believe that I have an appropriate level of expertise to confirm that it is of an acceptable scientific standard.
